# Planting seeds of change: reconceptualizing what people eat as eating practices and patterns

**DOI:** 10.1186/s12966-021-01102-1

**Published:** 2021-03-04

**Authors:** Dana Lee Olstad, Sharon I. Kirkpatrick

**Affiliations:** 1 Department of Community Health Sciences, Cumming School of Medicine, University of Calgary, 3280 Hospital Drive NW, Calgary, AB T2N 4Z6 Canada; 2 School of Public Health and Health Systems, University of Waterloo, 200 University Avenue West, Waterloo, ON N2L 3G1 Canada

**Keywords:** Dietary behaviors, Eating practices, Eating patterns, Socioecological model, Framing

## Abstract

Language focused on individual dietary behaviors, or alternatively, lifestyle choices or decisions, suggests that what people eat and drink is primarily a choice that comes down to free will. Referring to and intervening upon food consumption as though it were a freely chosen behavior has an inherently logical appeal due to its simplicity and easily defined targets of intervention. However, despite decades of behavioral interventions, population-level patterns of food consumption remain suboptimal. This debate paper interrogates the manner in which language frames how problems related to poor diet quality are understood and addressed within society. We argue that referring to food consumption as a behavior conveys the idea that it is primarily a freely chosen act that can be ameliorated through imploring and educating individuals to make better selections. Leveraging practice theory, we subsequently propose that using the alternative language of eating practices and patterns better conveys the socially situated nature of food consumption. This language may therefore point to novel avenues for intervention beyond educating and motivating individuals to eat more healthfully, to instead focus on creating supportive contexts that enable sustained positive dietary change. Clearly, shifting discourse will not on its own transform the science and practice of nutrition. Nevertheless, the seeds of change may lie in aligning our terminology, and thus, our framing, with desired solutions.

## Introduction

It is widely acknowledged that an array of varied and intersecting influences shape the foods and beverages individuals consume. These influences range from taste preferences, to the social and cultural meanings attributed to food, the availability and accessibility of food, to broader social norms and policies [1–4]. The role of influences external to individuals in structuring food and beverage ‘choices’ is explicitly recognized in the peer-reviewed literature and in government dietary guidelines which, for instance, emphasize the importance of communal meals and culturally-appropriate foods [5, 6]. Despite this recognition, the language ‘dietary behaviors’ is typically used to describe phenomena relevant to food consumption within health discourse, including within the peer-reviewed literature (ourselves not excepted) and policy-relevant documents [6, 7].

Language focused on individual dietary behaviors, or alternatively, lifestyle choices or decisions, suggests that what people eat and drink is primarily a choice that comes down to free will. Indeed, describing food consumption as a behavioral attribute connotes a straightforward and rational decision making process whereby individuals carefully weigh various considerations, such as taste, health, and economics, and calibrate their dietary selections accordingly [8]. Not only is this language inconsistent with the evidence regarding the automaticity of most food-related ‘decisions,’ [9] but more importantly, it downplays the contextual drivers that structure them, many of which reside outside the food system [10–12]. Given the focus on the individual rather than on broader socioecological influences, this language and the corresponding frames it invokes give precedence to interventions rooted in theories of behavior change to alter perceived deficits inherent within individuals [13, 14]. Moreover, this language and framing can perpetuate stigma by holding individuals responsible for any negative health outcomes that may ensue from consuming foods that are inconsistent with dietary guidelines [15].

Frames are mental structures that shape the way individuals see the world and their corresponding responses to information and events [16]. Frames overtly and covertly define problems and their causes, make moral judgments, and suggest solutions [17]. When frames become widely accepted, they may be regarded as self-evident truths [18]. Language is the primary means through which values and understandings are communicated within society, and is therefore a core aspect of framing [19–21]. Through shaping ‘frames in thought’, ‘frames in communication’ that are embedded in language play an integral role in determining the interventions we envision as most appropriate in responding to a given public health issue [19–21]. Given the power of frames and framing, public health actors have been urged to more actively *reframe* public health issues to promote desired solutions [22]. As suboptimal diet quality is a leading cause of global morbidity and mortality [23], the framing of food consumption warrants consideration in efforts to improve population health.

This debate paper interrogates the manner in which language frames how problems related to food consumption are understood and addressed within society. The specific objectives of this debate paper are to: 1) demonstrate that referring to food consumption as a behavior conveys the idea that it is primarily a freely chosen act that can be ameliorated through imploring and educating individuals to make better selections; and 2) propose that framing food consumption using the alternative language of eating practices and patterns may prompt a fuller accounting of the contextual drivers of food consumption, thereby promoting structural interventions that empower individuals to engage in healthier eating patterns. Although this paper focusses on nutrition-related discourse, the concepts discussed may be transferable to other health domains given that behavioral language is pervasive within many health disciplines.

### The language of dietary behaviors: an inadequate frame that focuses on individuals and their behavioral choices

The dominant positioning of food consumption as a behavior within health-related discourse is consistent with early theories of behavior change stemming from health psychology, many of which remain influential today [24, 25]. These theories posit a linear causal chain whereby individual psychological determinants, together with perceived social norms and environmental cues, shape food consumption [26]. Individuals are thereby regarded as free agents who are primarily cognitively motivated, and whose health-related actions can be improved by educating and motivating them to make better choices. Such linear thinking ignores the emergent and contingent nature of what people do within the contexts in which they are situated [26]. That is, rather than viewing individuals as complex social actors who are both influenced by, and act back upon, their social contexts, many behavioral theories conceive of individuals as dietary accountants who weigh the benefits and risks of particular dietary options and calibrate their selections accordingly [24, 27]. Within this framing, individuals are often regarded as accountable for food selection, and those who do not select optimally may be viewed as socially irresponsible and/or deficient in knowledge [13, 28]. Although we problematize use of the word ‘behavior’ in this paper, terms such as lifestyle, risk factors, and food choices can also reinforce an individualistic framing of food consumption.

Although some recent behavioral theories integrate contextual considerations (e.g. Michie et al’s [29] Behaviour Change Wheel), there remains a strong emphasis on individual behavior change within nutrition and other health disciplines [24, 25, 30]. For instance, clinical nutrition care often entails behavior change counselling and the prescription of tailored meal plans. Food-based dietary guidelines, even if they recognize some of the environmental drivers of food consumption, nevertheless tend to focus on individual behaviors. Similarly, there is no shortage of behavioral interventions within the health and nutrition literature, such as nutrition skill-building interventions, and many population-level nutrition policies, such as nutrition labelling and social marketing interventions, are predominantly informational and aim to help individuals make ‘more informed choices’ [31]. Such approaches may acknowledge the need to address certain factors external to individuals by, for example, teaching individuals how to navigate restaurant menus to make healthy choices and calling for changes to food environments. However, in the absence of coordinated approaches to create contexts that support healthy eating, many approaches to promoting healthy eating may inadvertently place the responsibility primarily on individuals, emphasizing their agency, autonomy and choice.

Language and corresponding frames that conceptualize, and thus intervene upon, food consumption as though it were a freely chosen behavior have an inherently logical appeal as they are relatively easy to articulate compared to more complex frames that recognize the array of factors that shape food consumption. This individualistic framing invokes easily defined targets of intervention [25] and fits well with popular notions of personal responsibility and free markets. By assuming that what people eat and drink is a behavior conditioned primarily by their deficient knowledge or motivation, interventions have only to educate or motivate individuals to choose differently. The affective, material, and relational aspects of food consumption that defy easy intervention can be largely ignored [26]. However, although well-supported behavioral interventions can be effective [32], evidence points to the inadequacies of individual-level interventions in improving food consumption patterns at a population level [33–36]. As such, despite decades of referring to and intervening on ‘dietary behaviors,’ societal patterns of consumption remain far from optimal [37–39]. Moreover, this behavioral framing has adverse social consequences, as evidenced by stigmatization of individuals who consume less healthful foods and beverages and those who live in larger bodies, with correspondingly negative implications for both physical and psychological health [15, 40–42]. Given that individuals who occupy lower social strata may face greater contextual barriers to maintaining healthy eating patterns [43], positioning food consumption as a behavior may be particularly damaging to efforts to reduce health inequities, further marginalizing already marginalized groups.

We are not the first to recognize the behavioral underpinnings of much of the current health-related discourse [44, 45]. Indeed, as Brady [45] recently pointed out, biomedical language is codified within the electronic Nutrition Care Process Terminology [45, 46], an internationally accepted standardized process and set of terminology to assess, diagnose, intervene upon and monitor nutritional concerns. Based on this review, Brady [45] concluded that the terminology prioritizes biomedical understandings of individuals’ nutritional concerns as being primarily related to deficiencies in individual knowledge, beliefs, and/or motivation [45]. Unfortunately, Brady [45] could find no language within this set of terminology that would allow dietitians to give voice to the socioeconomic realities of their clients’ lives.

Continuing to educate and implore individuals to eat more healthfully despite contextual conditions that overwhelmingly promote the opposite is unlikely to yield significant improvements in population-level patterns of consumption, nor in dietary inequities. However, the inadequacies of the behavioral paradigm have often been attributed to operational deficiencies within interventions themselves, such as suboptimal adherence or an inability to accurately measure change, rather than to inherent limitations of a narrow focus on individuals and their choices [14, 24, 26]. Language that frames eating as a behavioral choice further legitimates this narrow focus and restricts policymakers, practitioners, and researchers from both imagining and implementing more comprehensive, effective, and equitable approaches that engage with the broader structural factors that configure individuals’ eating practices and patterns [16, 44]. What is needed instead is a paradigm shift to better reflect the socially situated nature of individuals and their patterns of consumption, and to direct attention toward interventions that explicitly engage with the social, political, historical and economic realities of food consumption [44, 47].

### The language of eating practices and patterns: a comprehensive frame that invokes powerful contextual solutions

In contrast to the behavioral paradigm, a socioecological paradigm provides a framework for conceiving of individuals in recursive relationships with their environments [48]. It shifts the lens from individual attribution and personal responsibility to consider how individuals’ food consumption patterns form in relation to the opportunities and constraints imposed by their broader social contexts, including inter-personal relationships, community, and broader macro-social features, while also acknowledging the contribution of individual-level factors [49]. Particularly important are the societal institutions, policies, and norms that configure the conditions of daily life within which patterns of consumption develop and are reproduced. Thus, whereas a behavioral paradigm primarily invokes concepts of human agency, a socioecological paradigm reframes eating patterns as socially generated practices that reflect the embeddedness of individuals within particular contexts, integrating consideration of human agency and social contexts.

Theories of social practice have their origins in the work of Bourdieu, Foucault, Giddens, Marx, and others [50]. Although definitions are not apace, modern theorists commonly describe health practices as habitual health-related activities engaged in by collectives of individuals in a coordinated manner that emerge from, and are contingent upon, an array of individual and especially collective features [26, 50, 51]. Practices are always social and shared, persistent yet dynamic, and interact with others to form more complex bundles [50, 51]. Their continuance acts back to reshape existing social structures [4, 26, 50]. Put simply, health practices are the visible manifestations of the dynamic interaction between human agency and social contexts.

Reconceptualizing what people eat as socially generated eating practices and patterns rather than as behaviors more accurately reflects the duality of human agency and social contexts in shaping patterns of consumption [4]. Eating involves choices, but these choices are contingent upon the context in which they are made [4]. That is, people adopt particular eating patterns according to their assessments of what is structurally possible for them, for example, based on their socioeconomic position, access to the social determinants of health, and the social, cultural, political, and economic dimensions of their food environments [4, 52, 53]. Thus, the social context both empowers and constrains the exercise of human agency, and it is only by addressing contextual constraints that individuals can exercise their agency in health-promoting ways [49]. Over time, the dialectic interplay between social structures and human agency yields relatively stable eating practices and patterns that are reproduced over time and feed back to reinforce and/or modify the social context [4].

Shove et al. [54] describe practices as comprised of three interconnected elements: meanings (e.g. social expectations and symbolic meanings about how and why to perform a practice), materials (e.g. tools and objects needed to perform a practice), and competencies (e.g. the skills and knowledge needed to perform a practice). Practices persist when these three elements consistently come together and are accessible to individuals, and as new individuals are recruited to perform them [50, 54]. In this way, practices can become routines or habits [55, 56]. Crucially, it is in changing or breaking the connections between the three elements that the seeds of change ultimately lie, whereby practices can be disrupted and evolve, or fade and eventually disappear [50, 54]. Thus, rather than targeting the attitudes, behaviors and choices of individuals, practice theory regards practice elements as the unit of analysis and target of change [50, 55]. Individuals are thereby positioned as mere carriers of practices, and as the point where multiple practices intersect [57]. Given that materials are key resources that enable or constrain practices, they are particularly potent points of intervention [50]. Moreover, when connections form between practices (e.g. snacking and watching television), changes in one practice can be leveraged to disrupt another [50, 55].

Applied to the practice of eating breakfast, a nutrition professional might consider how to redirect the three elements of materials, meanings, and competencies to support healthful consumption patterns [50]. For instance, interventions could focus on altering the meanings of breakfast (e.g. social norms as to what foods it should include), the availability of materials (e.g. access to and cost of healthy food), and on enhancing competencies (e.g. cooking skills) [50]. Snacking practices could similarly be addressed by seeking to alter the meanings of snacking (e.g. a healthful supplement to meals), materials (e.g. the availability of healthful items within vending machines) and competencies (e.g. knowledge of convenient sources of healthful snacks) [55]. Notably, however, from a socioecological perspective, there is also an inherent ordering to these elements, as without the structural and social supports provided by health promoting materials and meanings, altering individual competencies will do little to disrupt habitual eating practices.

Conceiving of patterns of food consumption as collective practices influenced by factors at multiple socioecological levels, rather than as cognitively motivated behaviors performed by disconnected individuals, also points to the embeddedness of eating patterns within global food systems that produce, manufacture, market, and sell predominantly highly processed, inexpensive, palatable, and convenient foods. On the one hand, these food systems are part of what configures the unhealthy eating practices of individuals through shaping the social meanings of food consumption (e.g. via food marketing) and the availability of requisite materials (e.g. widespread availability of energy-dense, nutrient-poor foods), whereas on the other, the macro-level activities that result in the overproduction of unhealthy foods are themselves sets of practices shaped by social practices at other levels (e.g. dual-income families, busy lifestyles) and within other sectors. Thus, individuals’ eating patterns are recursively configured by many types of practices, and these practices are all interlinked [58]. Framing eating patterns in this manner, as an emergent property of social relations across multiple levels and sectors, has profound implications for how they can be transformed. Strategies that focus on changing the ‘dietary behaviors’ of individuals who are positioned at the end of this long chain of social relations are unlikely to prove as effective as those that explicitly acknowledge and engage with these interdependencies by disrupting practices at higher levels.

Thus, whereas a behavioral paradigm delimits relatively narrow opportunities for intervention focussed on individual agency, language that invokes eating practices and patterns better conveys the socially situated nature of food consumption and broadens the scope for intervention beyond individuals and their cognitions. Framing food consumption using the language of eating practices and patterns suggests novel avenues for intervention beyond educating and motivating individuals to eat more healthfully, to instead focus on creating supportive contexts (e.g. by altering social meanings and materials) that enable sustained positive change. Such language may foster greater recognition of the social and cultural meanings of food, and create distance from harmful diet culture and ‘dieting’ efforts [59], which are inconsistent with healthy eating patterns. It also avoids blaming and shaming individuals, who are in fact acting in accordance with the socioeconomic and other circumstances that confront them. Similar language and frames may be applicable to other health domains to describe practices related to physical activity, sleep, tobacco consumption, and others.

The preceding discussion is not intended to discount the importance of behavioral interventions under appropriate circumstances, nor to absolve individuals from some level of personal responsibility. Rather, we are appealing for a middle ground that uses more inclusive language to frame eating and other health-related practices not simply in biomedical terms, but also as social phenomena. Just as society no longer labels individuals in terms of disease states (e.g. a diabetic individual versus an individual with diabetes), so too should individuals no longer be characterized as having freely chosen to engage in particular dietary behaviors, but rather as practitioners of eating practices and patterns that reflect their contextual opportunities and constraints. By resisting the search for direct, causal antecedents, and instead giving expression to the dialectic interplay between human agency and social structures, this language may also better support the translation of socioecological understandings of eating and health into research, practice, and policy.

### Shifting nutrition-related discourse: a way forward

Although adopting the language of eating practices and patterns may appear to be a simple proposal, in reality, it will require a broader project of change. Language and frames are underpinned by epistemologies that are deeply embedded within the culture of most health professions and are reinforced by professional training programs, socialization, roles and norms that accord with biomedical perspectives [60, 61]. Broader structural forces (e.g. government health budgets) further entrench such perspectives by according primacy to clinical nutrition care delivered in healthcare settings. Therefore, while shifting language to frame eating as an emergent social practice can certainly provoke important conceptual shifts, if we wish to truly transform nutrition-related discourse, we must simultaneously challenge the epistemologies that inform it.

An insightful analysis by Brasolotto et al. [60] provides a case in point. The authors identified three clusters of public health units in Ontario, Canada based on the language they used to discuss, and thereby frame, the social determinants of health. The authors found that the manner in which the health units acted to address the social determinants of health in practice reflected their discursive patterns. In other words, public health units enacted their discourse. Public health units in which staff discussed the structural origins of the social determinants of health were distinct from all of the others in that they alone sought to intervene at the most upstream levels to reduce health inequities. These findings highlight how health-related epistemologies inform language, frames, and action.

A feasible and logical starting point may therefore be to reorient training programs and competencies related to dietetics and nutrition towards a greater focus on equipping nutrition professionals with the knowledge and skills to address the social (i.e. meanings surrounding food consumption, such as social norms) and structural constraints (i.e. materials to support healthful eating patterns, such as sufficient income) that individuals and populations face in maintaining healthful dietary patterns. Such a reorientation would help to ensure that unhelpful biomedical epistemologies and discourses do not become entrenched in the first place, and would also provide the opportunity to instill the language of eating practices and patterns early on. In particular, coursework and experiential learning should provide greater exposure to the social sciences (e.g. anthropology, sociology), non-positivist research methodologies (e.g. ethnography), and foundational public health principles (e.g. health equity, socioecological models). Once equipped with foundational knowledge and skills, professional roles could be rebalanced away from their current emphasis on the management of acute conditions and chronic disease in clinical settings, to ensure a much greater presence of nutrition professionals within communities and at the highest levels of policy making. Such a rebalancing of professional roles would require redirecting a greater share of healthcare spending towards public health, and a commitment to health in all policies; changes that are not impossible but that will certainly require sustained efforts.

There is evidence that some of the changes we have advocated herein are underway. For instance, Brady has developed nutrition justice terminology for dietetic practice [45]. Hughes et al. [62] have outlined a comprehensive list of evidence-based competencies for dietitians working in public health and community nutrition settings. There is a large body of research on food environments and policies, and many researchers are investigating programs and policies to improve the healthfulness of food environments, emphasizing the contextual determinants of eating practices [63]. Moreover, many social scientists are investigating snacking and meal patterns as socially situated practices rather than as freely chosen behaviors [4, 50, 55, 64]. Ultimately, we expect that increasing recognition of food consumption as a socially situated practice will promote more socially responsive nutritional care for individuals and populations that will advance dietary and health equity.

### Summary and conclusions

Unhealthy patterns of food consumption are pervasive and seemingly intractable. However, their continuance is unintentionally supported by language that implies a separation of individuals from their environments and that exaggerates the extent to which rational choice drives food consumption. The power of language stems from the frames it invokes, as frames lead individuals to conceive of the causes and solutions to health-related problems in a particular manner. By framing what people eat as an individual behavioral choice, the language of dietary behaviors delimits relatively narrow and overly simplistic avenues for intervention focussed on altering factors internal to individuals. By contrast, the language of eating practices and patterns invokes a socioecological frame that regards individuals’ food consumption practices as enmeshed within their broader social contexts. This language conveys the idea that individual-level interventions represent but one component of broader, multi-level approaches that are essential to improve collective patterns of consumption. Changing nutrition-related discourse will take time, and will require altering epistemological understandings of the contingent and emergent nature of eating practices and patterns and of their social and structural determinants. Nevertheless, the scale of the challenge should not preclude action. The ultimate aim of these epistemological and discursive shifts is to enhance attention to the contextual origins of eating patterns, and stimulate corresponding contextual interventions that enable individuals to exercise their agency in health promoting ways. Such interventions may have a greater likelihood of success than individualistic approaches because they reach a substantial proportion of the population and address root causes of unhealthy eating patterns rather than their behavioral symptoms. By addressing contextual barriers that constrain the eating patterns of disadvantaged groups, these interventions may also help to reduce dietary and health inequities.

Words carry meaning and consequences [44]. Thus, the seeds of change may lie in aligning our language, and thus, our framing, with desired solutions. Clearly, shifting health discourse will not on its own transform the science and practice of nutrition. Nevertheless, it is an important step. Nutrition and other health experts, particularly those from the social sciences, are ideally placed to drive a paradigm shift away from a behavioral and towards a socioecological framing of food consumption.

## Data Availability

Not applicable.
